# Longitudinal Presentation and Management of Neutrophilic Dermatosis of the Dorsal Hand: A Case Report Over Six Visits

**DOI:** 10.7759/cureus.68128

**Published:** 2024-08-29

**Authors:** Matthew H Bohman, Hunter Kall, Andrew Miner

**Affiliations:** 1 Dermatology, Burrell College of Osteopathic Medicine, Las Cruces, USA; 2 Dermatology, Brevard Skin and Cancer Center, Rockledge, USA

**Keywords:** sweet syndrome, autoinflammatory disorder, acute febrile neutrophilic dermatosis, neutrophilic dermatosis, gomm-button disease

## Abstract

Neutrophilic dermatosis of the dorsal hands (NDDH) is a rare, challenging condition characterized by its acute febrile onset, neutrophilic dermal infiltrate seen on histology, and frequent association with hematologic disorders. This case report presents a longitudinal follow-up of NDDH in an 80-year-old male with significant comorbid conditions, including atrial fibrillation, chronic systolic heart failure, venous insufficiency, and gastroesophageal reflux disease. Over six clinic visits, we document the patient's clinical progression, highlighting key changes in presentation, diagnostic findings, and the multifaceted approach to management, including the use of systemic and topical therapies. Notably, the treatment strategy was adapted in response to diagnostic tests revealing methicillin-resistant *Staphylococcus aureus* (MRSA) and Group B *Streptococcus* (GBS), complicating the patient's condition. Despite the challenges posed by the patient's comorbidities, which limited the use of certain medications, the patient showed significant improvement with a treatment approach primarily involving topical and systemic steroids. The observed improvement emphasizes the effectiveness of modifying therapy based on lesion regression and the patient's overall condition. This case underscores the critical need for accurate diagnosis, the complexities of managing NDDH in patients with multiple health conditions, and the value of adaptability in treatment plans. Through this detailed longitudinal case study, we contribute insights into the progression and management of NDDH, emphasizing the need for ongoing research and a flexible approach to treatment.

## Introduction

Neutrophilic dermatosis of the dorsal hands (NDDH) is described as a rare variant of Sweet syndrome (acute febrile neutrophilic dermatosis) that is localized to the upper extremities, with a distribution that is exclusively on the dorsal aspect of the hands [1]. NDDH presents clinically as an abrupt onset of tender erythematous plaques or nodules on the dorsal hands. NDDH resembles subsets of vasculitic or infectious processes due to widespread ulceration, pustule formation, or central necrosis of the lesion, which often leads to frequent misdiagnosis. Severe cutaneous manifestations of neutrophilic dermatosis can be put into the subset "necrotizing neutrophilic dermatosis" due to the resemblance with necrotizing fasciitis. A case series reported that these instances of necrotizing neutrophilic dermatosis were initially misdiagnosed as necrotizing fasciitis 94% of the time [2]. Although an estimated 50-71% of Sweet syndrome cases have an idiopathic (classic) origin seen with concurrent conditions like inflammatory bowel disease, 21% of cases can be seen as a paraneoplastic lesion from underlying hematologic or solid tumor malignancy [3]. The use of antibiotics or surgical debridement with amputation is typically unnecessary and may delay the treatment of the underlying disease causing the skin manifestation [4]. Topical or oral glucocorticoids are the mainstay of therapy to treat Sweet syndrome and NDDH, while dapsone and methotrexate have also been employed as steroid-sparing agents with some promising results [5].

## Case presentation

Patient background

An 80-year-old man presented to the clinic with painful, non-healing, ulcerated lesions on the dorsum of both of his hands that have persisted for the past 10 months. A review of systems was negative for unintentional weight loss, night sweats, hematochezia, gastrointestinal symptoms, or history of pathergy. Past medical history was significant for atrial fibrillation, chronic renal insufficiency, anemia, chronic systolic heart failure, venous insufficiency, and gastroesophageal reflux disease.

Visit 1: November 2022

Clinical Observations

**Figure 1:**
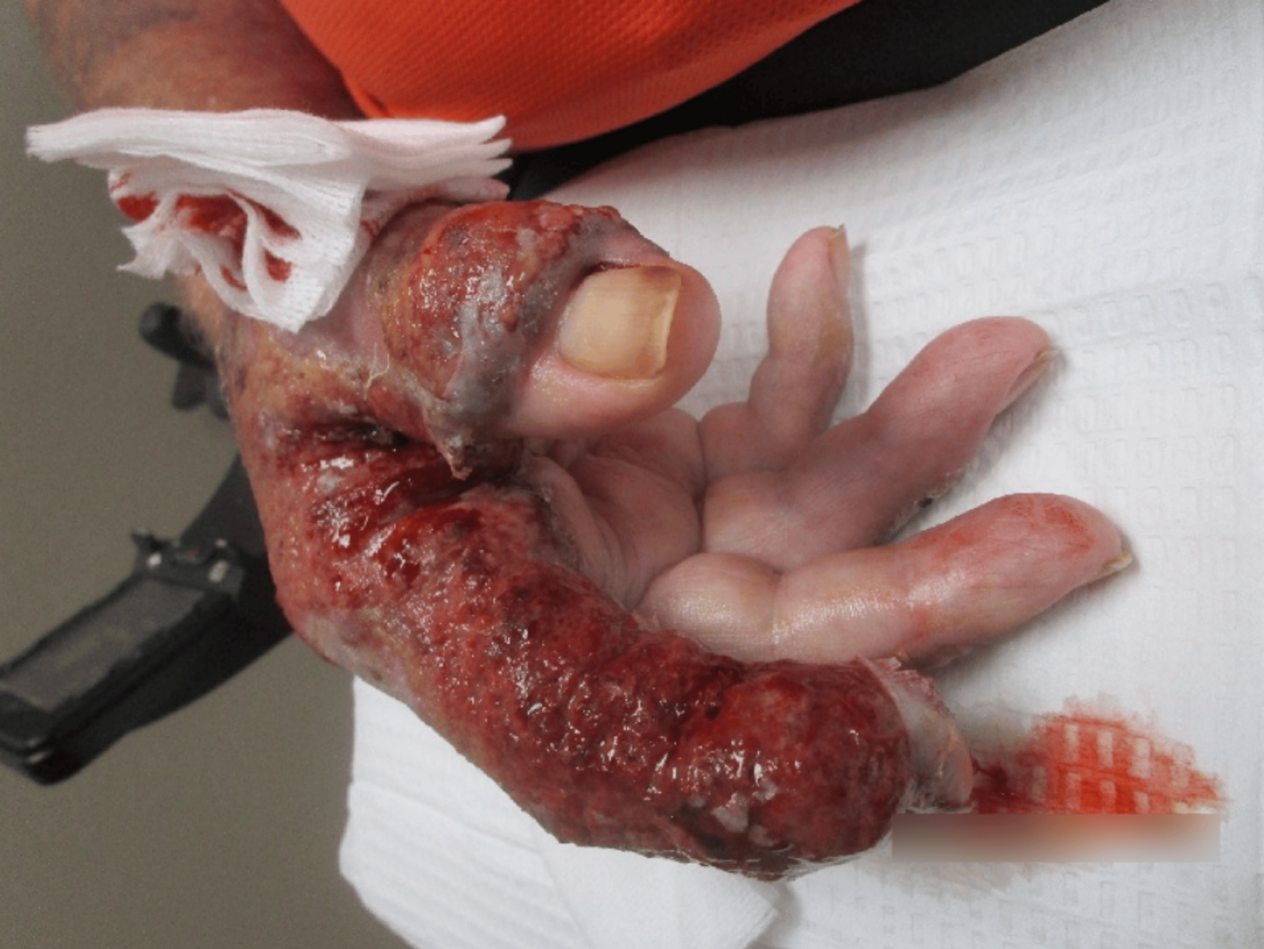
Right hand at the first clinical visit, displaying extensive, ulcerated, erythematous, and violaceous plaques with serosanguinous exudate and peripheral crusting of the first and second digits.

**Figure 2:**
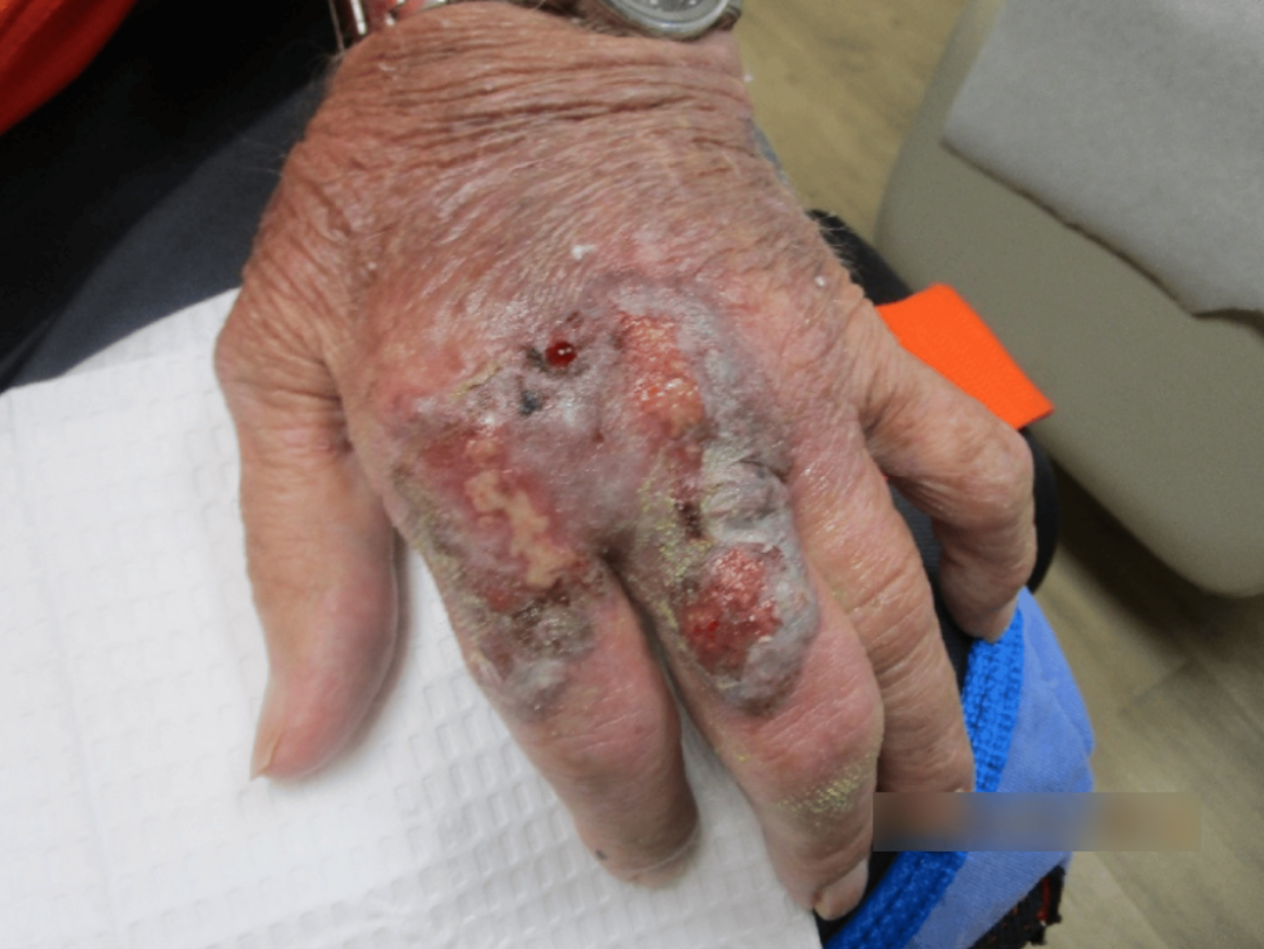
Left hand at the first clinical visit, displaying ulcerated erythematous and violaceous plaques with serosanguinous exudate, peripheral crusting, and focal hemorrhage, primarily involving the dorsal aspects of the second and third digits.

Examination revealed erythematous and violaceous plaques limited to the dorsal hand, characterized by a mixed hue ranging from dusky red to brownish-black. The surface of the plaques consisted of a heterogeneous mix of crusting and scaling, indicative of varying stages of lesion evolution. These areas of ulceration displayed a moist, yellowish base with serosanguinous drainage and erythematous, well-demarcated margins bordering peripherally crusted portions of the lesion (Figure 1 and Figure 2).

Diagnostic Tests

A 4 mm punch biopsy was taken from the dorsum of the right hand to rule out NDDH and Sweet syndrome, pyoderma gangrenosum, acrodermatitis continua of Hallopeau, pustular psoriasis, and mycobacterial, viral, and fungal infections.

Visit 2: November 2022, two days later

Clinical Observations

There was no change in presentation since the initial visit.

Diagnostic Tests and Results

**Figure 3:**
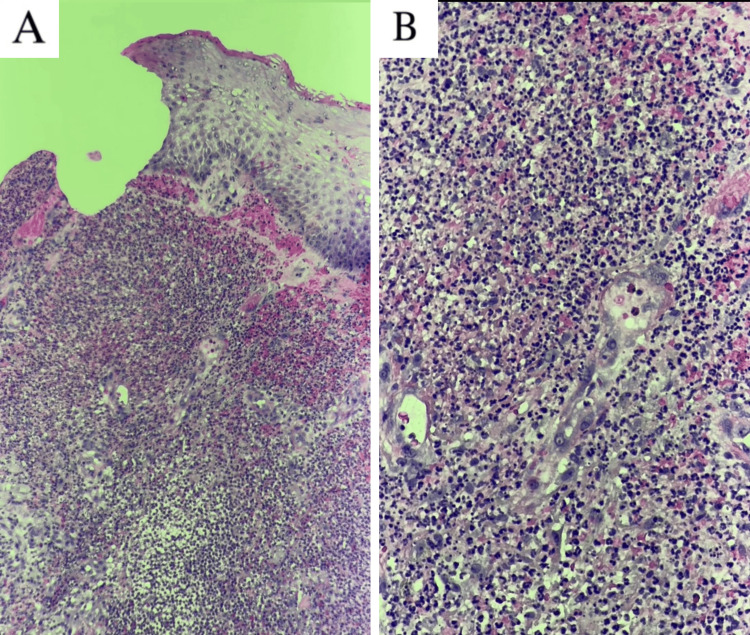
Punch biopsy results showing dense neutrophilic infiltrate on hematoxylin and eosin staining at (A) 40× magnification and (B) 100× magnification.

Cutaneous bacterial cultures were taken from the lesions on both hands to test for concomitant infection. A punch biopsy of the right hand showed a dense neutrophilic infiltrate with leukocytoclasia, without evidence of vasculitis, consistent with NDDH (Figure 3).

Treatment Administered

The patient was prescribed clobetasol propionate 0.05% and mupirocin ointment 2% to address both the punch biopsy results and the risk of concomitant bacterial infection. Concurrently, the patient was started on a tapered oral prednisone prescription over 20 days total, beginning at 40 mg (0.5 mg/kg). 

Visit 3: November 2022, one week later

Clinical Observations

**Figure 4:**
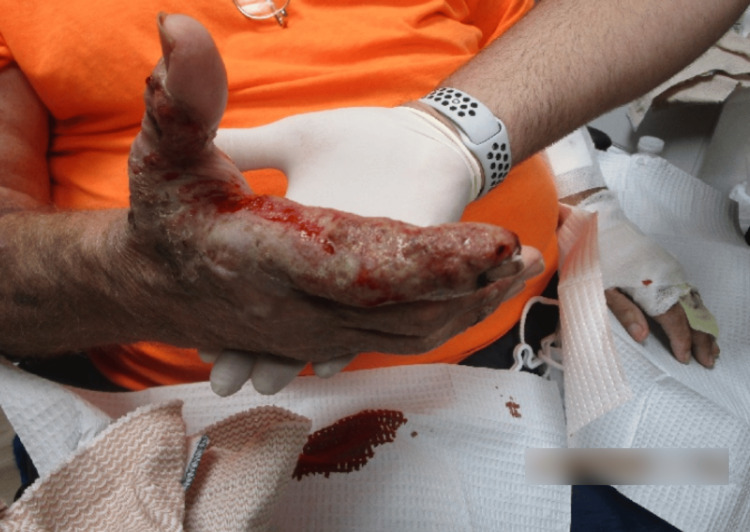
Right hand one week after the initial treatment, displaying erythematous, ulcerated plaques with serosanguinous exudate and peripheral re-epithelialization.

**Figure 5:**
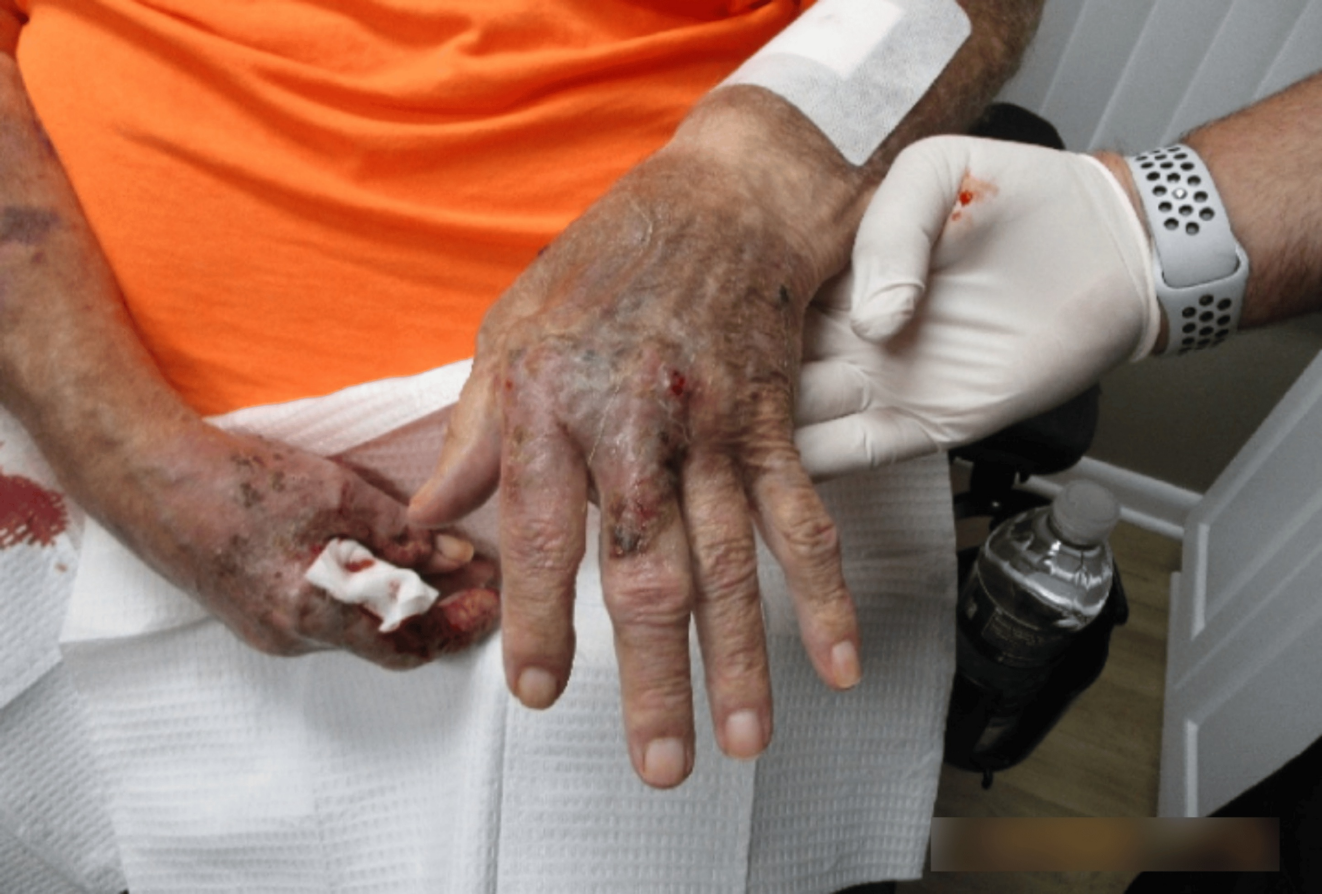
Left dorsal hand one week after the initial treatment, showing nascent skin with focal areas of residual ulceration and peripheral crusting of the third MCP and the proximal portion of the third digit. MCP: metacarpophalangeal

Upon examination, the patient's bilateral hand lesions had considerably improved since the last office visit five days prior. The previously noted ulcerated, serosanguinous lesions persisted, yet appeared less inflamed and reduced in size. The erythema was less pronounced, and the lesions exhibited early signs of re-epithelialization. There was no evidence of new lesion formation or spread to other areas. This marked improvement in clinical findings suggested a positive response to the prescribed treatment regimen. Continued monitoring and follow-up were planned to ensure ongoing healing and to manage any potential recurrence or complications (Figure 4 and Figure 5).

Diagnostic Tests and Results

The cultures obtained at the previous visit from the ulcerated lesions on the patient's hands yielded positive results for methicillin-resistant *Staphylococcus aureus* (MRSA) and Group B *Streptococcus* (GBS).

Adjustments to Treatment

To address the identified pathogens, gentamicin sulfate 0.1% topical ointment was added to the pre-existing treatment regimen. Gentamicin was chosen for its efficacy against both MRSA and GBS. Furthermore, the treatment with clobetasol propionate 0.05% and oral prednisone was continued to manage the inflammatory aspect of the dermatosis.

Visit 4: November 2022, two weeks later

Clinical Observations

Since the last office visit, there has been further improvement in the patient's dermatological condition. Upon examination, the lesions previously observed on the left hand have undergone complete regression, revealing newly epithelialized skin. This nascent skin appears healthy, with a pink hue, and exhibits characteristics of post-inflammatory regeneration. On the right hand, the lesions demonstrate marked improvement, with their presence now confined to the volar aspect of the thenar eminence and the lateral aspects of the first and second digits. The area exhibits signs of healing with reduced erythema and swelling, indicating a visibly reduced inflammatory response. 

**Figure 6:**
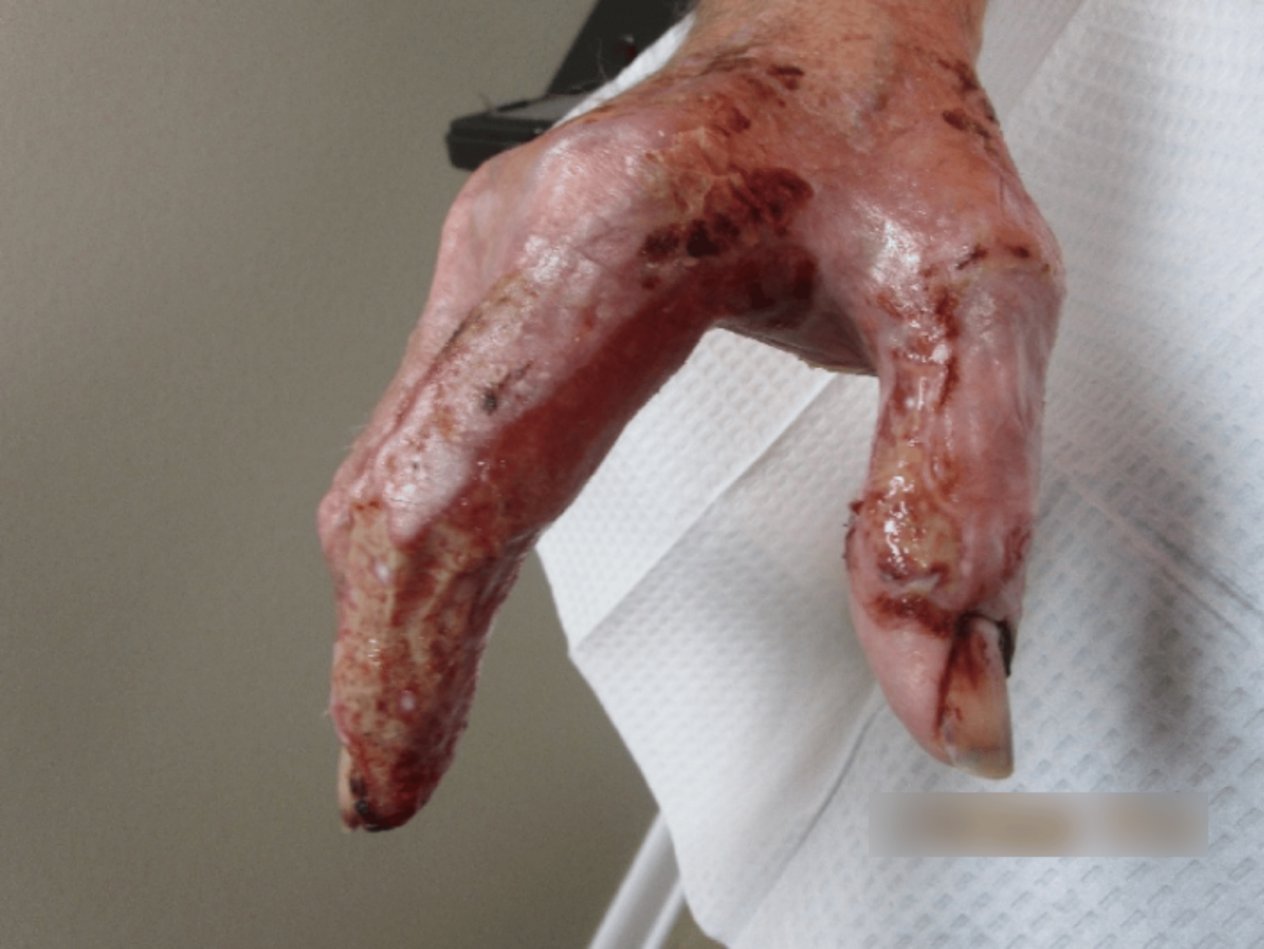
Right hand two weeks after the initial treatment, demonstrating re-epithelialization with a marked reduction in erythema and ulceration. The lesions show a fibrinous base with minimal residual crusting, and the skin appears smoother with decreased exudate, particularly along the second digit.

**Figure 7:**
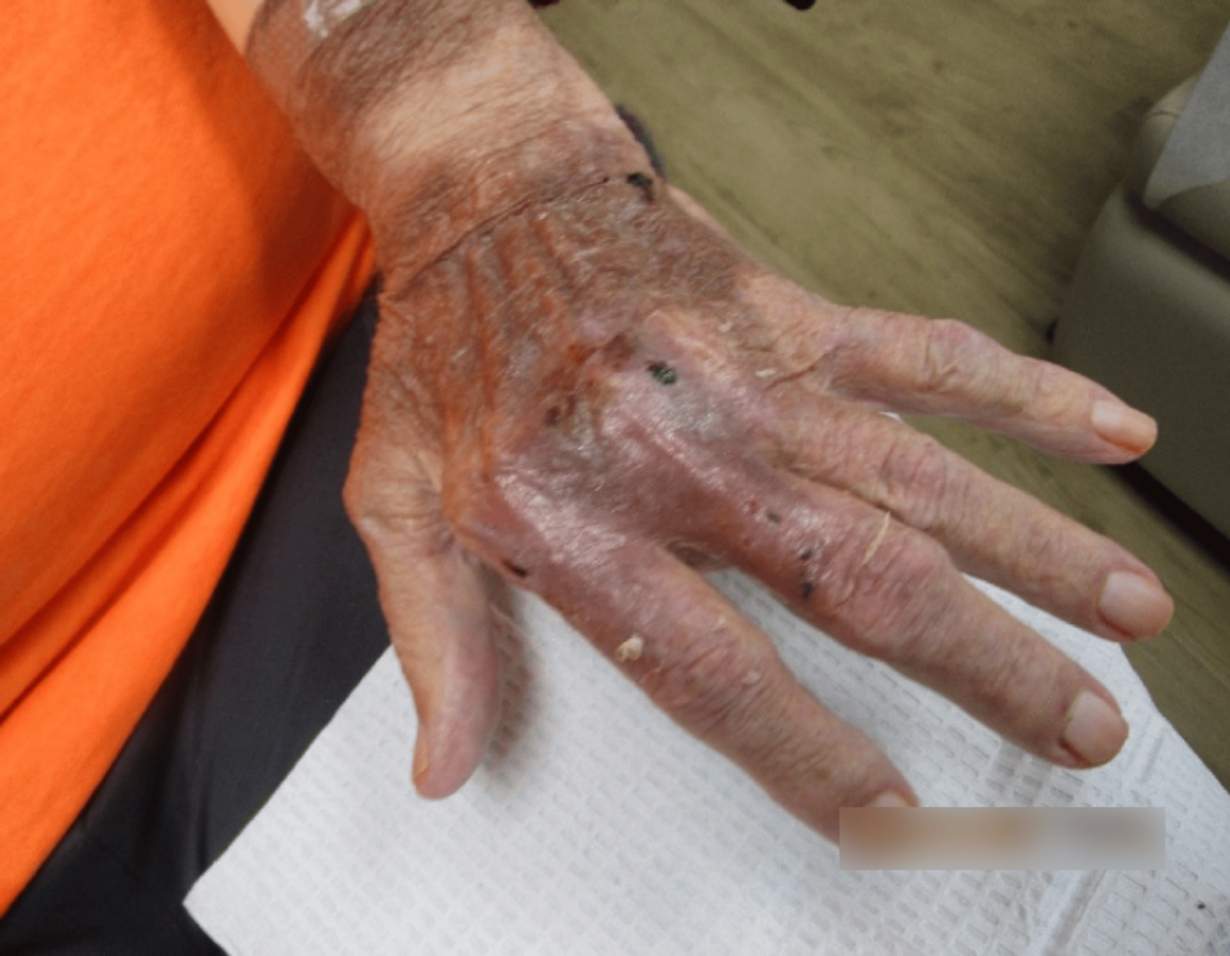
Left hand two weeks after the initial treatment, displaying nascent skin with hyperpigmentation and mild erythema. Minimal ulceration remains with scant crusting, primarily over the dorsal aspect of the third digit.

The areas where lesions have resolved are characterized by the absence of crusting, exudation, or open wounds, indicating effective re-epithelialization. There is no evidence of secondary infection or complication in the newly formed skin (Figure 6 and Figure 7).

Diagnostic Tests and Results

In order to evaluate for a potential underlying hematologic pathology, the following laboratory tests were ordered: complete blood count (CBC), glucose-6-phosphate dehydrogenase (G6PD) levels, serum protein electrophoresis (SPEP), iron studies (FE), and ferritin levels.

Treatment Administered

In light of the patient's prior medical history and favorable response to prednisone and clobetasol, the current therapy was maintained with a 10-day prolongation of oral prednisone, dosed 10 mg once daily.

Visit 5: November 2022, three weeks later

Clinical Observations

**Figure 8:**
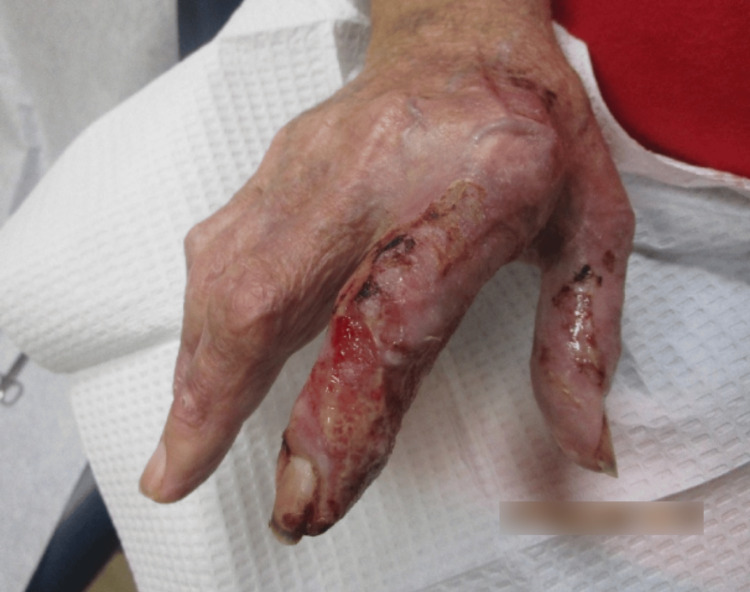
Right hand three weeks after the initial treatment, exhibiting nascent skin. Residual ulceration with a fibrinous base is present along the dorsolateral aspect of the second digit and the medial portion of the first digit.

**Figure 9:**
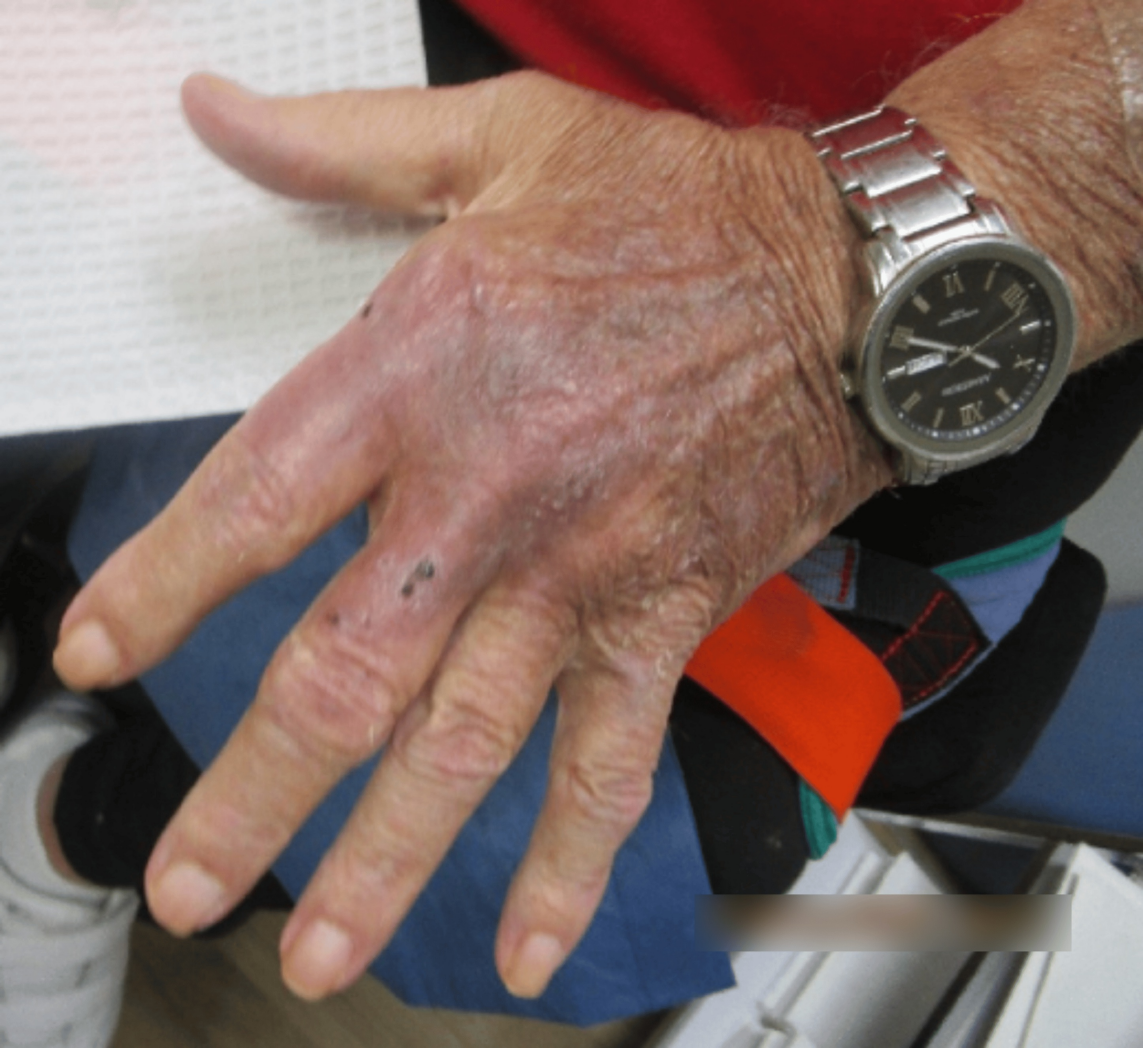
Left hand three weeks after the initial treatment, exhibiting nascent skin with areas of hyperpigmentation along the dorsal aspect, extending over the first, second, and fifth digits.

The patient has demonstrated continued improvement in their dermatological condition. Further regression of the lesions on the right hand was noted, particularly with the presence of healthy, re-epithelialized skin that is tight, is smooth, and exhibits a pink hue indicative of ongoing post-inflammatory healing. The areas of concern now exhibit markedly reduced erythema and swelling (Figure 8 and Figure 9).

Diagnostic Tests and Results

The laboratory tests ordered previously remain pending at this time. The positive response to treatment and delay in obtaining these results necessitates the continuation of the current treatment strategy, with a careful review planned once the laboratory data is available.

Visit 6: November 2022, four weeks later

Clinical Observations

**Figure 10:**
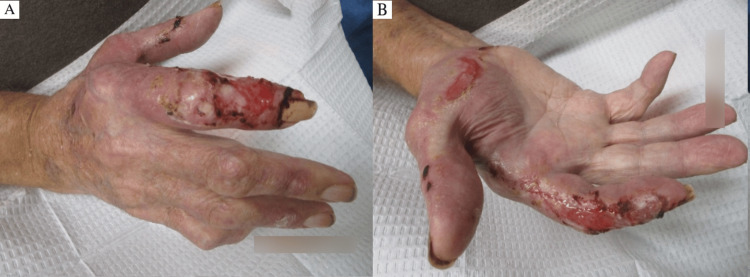
Right hand four weeks after the initial treatment. (A) Nascent skin and residual ulceration along the dorsolateral aspect of the distal interphalangeal joint, with peripheral crusting of the second digit phalanx. (B) Nascent skin with residual ulceration along the thenar eminence and dorsolateral aspect of the distal phalanx of the second digit, with peripheral crusting.

On evaluation, ongoing clinical improvement was evident, with the lesions across the patient's right hand demonstrating continued healing, nearing a 90% overall improvement since their initial presentation. However, the second digit of the right hand exhibited a slight increase in swelling compared to the previous week, drawing attention to the persistent challenge in fully resolving the patient's condition. The affected area, particularly the lateral portion of the distal interphalangeal (DIP) and intermediate phalanx of the second digit of the right hand, retained ulcerations that have yet to achieve complete re-epithelialization. These lesions, while significantly reduced in severity, still displayed a degree of inflammation and were in the process of healing (Figure 10).

Diagnostic Tests and Results

The laboratory tests ordered on the November 16, 2022, visit remain pending at this time.

Treatment Administered

The persistence of specific lesions, particularly on the second digit of the right hand, and the limitations posed by the patient's anemia and decreased renal function necessitated the following treatment modifications: The dosage of prednisone was increased from 10 mg once daily (QD) to 20 mg QD to bolster the anti-inflammatory response and facilitate further healing of the ulcerated regions. Colchicine was introduced at a dosage of 0.6 mg twice daily (BID) for an initial period of one week. This decision was grounded in colchicine's proven efficacy in modulating inflammatory responses and its decreased propensity to exacerbate pre-existing renal impairment. The patient will be closely monitored for any adverse effects, such as gastrointestinal symptoms, with the possibility of increasing the dose to 1.2 mg BID, based on tolerance. Previous wound care protocols, antibiotic treatment, and clobetasol application were maintained.

Follow-up and outcomes

Throughout the six visits documented in this case report, the patient demonstrated marked clinical improvement under the prescribed treatment regimen, with near-complete regression of the lesions. The management strategy, including the use of systemic and topical therapies, aimed to address both the inflammatory nature of NDDH and any secondary infections. Unfortunately, the patient did not return for additional follow-ups or complete the ordered laboratory tests and was later reported to have passed away. The cause of death and its potential relation to NDDH or the underlying conditions could not be determined due to the lack of further clinical interaction and diagnostic outcomes.

## Discussion

This case report outlined the resolution of NDDH over six visits, highlighting the variability in the disease's clinical course. Neutrophilic dermatosis typically presents with cutaneous hallmark features consisting of tender and irregular violaceous plaques, nodules, and pustules that can manifest as widespread ulceration or even necrosis as the disease goes untreated [6]. However, the nuances in its progression, associated systemic pathology, and variable response to treatments underscore the condition's complexity as a whole. Potassium iodide, topical plus oral corticosteroids, and colchicine have been regarded as the mainstay of therapy to treat neutrophilic dermatosis, but second-line options like dapsone and cyclosporine have been employed for recurrent cases with considerable degrees of success [7]. The use of systemic corticosteroids typically results in the substantial resolution of symptoms within 48 hours and can aid in diagnosis, as malignancy-associated cases of NDDH are often recalcitrant to treatment [8]. Moreover, a review found that 21% of patients with Sweet syndrome were diagnosed or would later be diagnosed with a solid or hematologic malignancy [9,10]. 

Recent research has highlighted possible inflammatory mechanisms underlying neutrophilic dermatoses, involving cytokines such as IL-1, IL-17, and IL-36. Dysregulation of the IL-1 pathway, crucial in mediating inflammatory responses, is a prominent feature in these conditions. IL-17 contributes to neutrophil activation and recruitment, while IL-36 amplifies the inflammatory network by enhancing IL-1α levels. These cytokines play significant roles in the pathophysiology of conditions like generalized pustular psoriasis and potentially other neutrophilic dermatoses [11]. The rationale behind the treatment regimen, particularly the use of corticosteroids and colchicine, was informed by the current understanding of NDDH's autoinflammatory pathophysiology.

## Conclusions

This case report provides valuable insights into the management of NDDH, highlighting the challenges of treating this condition in the context of co-existing medical issues. In this case, compromised renal function precluded the use of additional therapies that may have aided in the complete resolution of the lesion. The patient's concurrent cutaneous bacterial infection necessitated adjustments to the treatment protocol and underscores the importance of identifying concomitant factors affecting the lesion. The case also emphasizes the critical need for patient engagement in the treatment process to monitor treatment efficacy and exclude underlying hematologic conditions that could contribute to the disease. While there was significant improvement in the patient's clinical presentation, the incomplete resolution of symptoms suggests there is still room for enhancing treatment strategies for NDDH.

For clinicians managing similar cases, this report advises vigilance for potential microbial infections and underlying pathology contributing to NDDH, as well as considering the broader implications of systemic treatments in patients with complex medical profiles.
